# Analytical methods used to quantify isoflavones in cow’s milk: a review

**DOI:** 10.1007/s13594-015-0276-8

**Published:** 2016-01-13

**Authors:** Frédéric Daems, Jean-Michel Romnee, Stéphanie Heuskin, Éric Froidmont, Georges Lognay

**Affiliations:** Valorisation of Agricultural Products Department, Walloon Agricultural Research Center, Chaussée de Namur 24, B-5030 Gembloux, Belgium; Agro-Bio Chem Department, Gembloux Agro-Bio Tech, University of Liège, Passage des Déportés 2, B-5030 Gembloux, Belgium; Production and Sectors Department, Walloon Agricultural Research Center, Rue de Liroux 8, B-5030 Gembloux, Belgium

**Keywords:** Isoflavones, Phytoestrogens, Milk, Quantification, Analysis

## Abstract

This paper provides an update and comprehensive review of the analytical methods used for quantifying isoflavones and their metabolites in cow’s milk. Isoflavones are secondary plant metabolites that are similar to 17 β-estradiol in chemical structure. They form one of the most common categories of phytoestrogens. Numerous health benefits have been attributed to isoflavones, but many of these compounds are also considered to be endocrine disruptors, with adverse effects on health. These contradictory trends offer an attractive prospect for future research, and therefore, sensitive and reliable analytical methods are required to clarify various issues about isoflavones. For this review, a structured methodology was used to select 26 relevant articles published between 2005 and 2015 from the Scopus and CAB Abstract databases. The review discusses individual steps of the analytical procedures described in these articles, including sample preparation, instrumental analysis and validation. The most commonly used analytical procedure is sample preparation involving liquid-liquid extraction and an enzymatic hydrolysis step followed by liquid chromatography with mass spectrometry analysis. Currently, however, there is no standardized procedure for the sample preparation and analysis of isoflavones in milk.

## Introduction

**Fig. 1:**
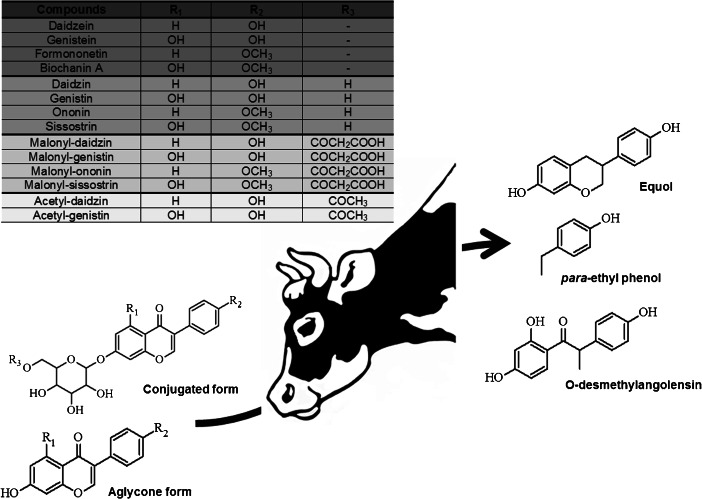
Chemical structures of some of the main isoflavones occurring in forages and their microbial metabolites found in milk (Kalač 2013; Vitale et al. 2013; Mostrom and Evans 2012; Saviranta et al. 2010)

Isoflavones are classified as phytoestrogens, which are widely distributed in the plant kingdom. These plant secondary metabolites are structurally similar to 17 β-estradiol and bind selectively, but weakly, to mammalian estrogen receptors (ER), with a preference for ERβ (Vitale et al. 2013; Baber 2013; Mostrom and Evans 2012). These diphenolic compounds appear to have antioxidant activity and could offer alternative therapies for a range of hormone-dependent conditions, including cancer, menopausal symptoms, cardiovascular disease, and osteoporosis (Ko 2014; Vitale et al. 2013; Baber 2013; Mostrom and Evans 2012; Mortensen et al. 2009). They also can be considered as endocrine disruptors, however, with the potential to have adverse health effects (Wielogorska et al. 2015; Sirotkin and Harrath 2014; Maggioni et al. 2013; Wocławek-Potocka et al. 2013; Patisaul and Jefferson 2010; Afssa 2005). The highest concentrations are found in plants of the Fabaceae family, such as soybean (*Glycine max* L.) and clover (*Trifolium pratense* L.) (Ko 2014; Mostrom and Evans 2012). In plants, isoflavones are usually found in conjugated forms as glucosides, acetylglucosides, and malonylglucosides (Kalač 2013; Mostrom and Evans 2012). When ingested by cows, isoflavones appear to be metabolized mainly in the rumen, and the main route of excretion is through feces and urine, with only a small proportion being excreted in milk (Njåstad et al. 2014). A diagram of isoflavone metabolization in cows is shown in Fig. 1. Biochanin A (BA) is demethylated mainly into genistein (GE) and via a ring cleavage into *para*-ethyl phenol (a compound that apparently has no estrogenic activity) and organic acids (Njåstad et al. 2014; Kalač 2013; Mostrom and Evans 2012). Formononetin (FO) is demethylated into daidzein (DA) and then reduced via hydrogenation and ring scission to equol (EQ) (a microbial metabolite of isoflavone with high estrogenic activity). FO can also be metabolized into other metabolites, such as *O*-desmethylangolensin (*O*-DMA) (compounds with low estrogenic activity) (Njåstad et al. 2014; Kalač 2013; Mostrom and Evans 2012; Setchell and Clerici 2010). The aglycones seem to be the most biologically active forms and are quickly absorbed by the rumen and gut mucosa (Vitale et al. 2013; Kalač 2013; Mostrom and Evans 2012; Lundh 1990). Most of them are conjugated by glucuronic acid (a limited amount is conjugated with sulfate) during this absorption process. A small proportion of aglycones reaches the blood circulatory system, but the aglycones are rapidly conjugated in the liver and other tissues (Mostrom and Evans 2012; King et al. 1998; Lundh 1990). These isoflavone metabolites then circulate throughout the body and are excreted in feces and urine or transferred to the milk.

**Fig. 2:**
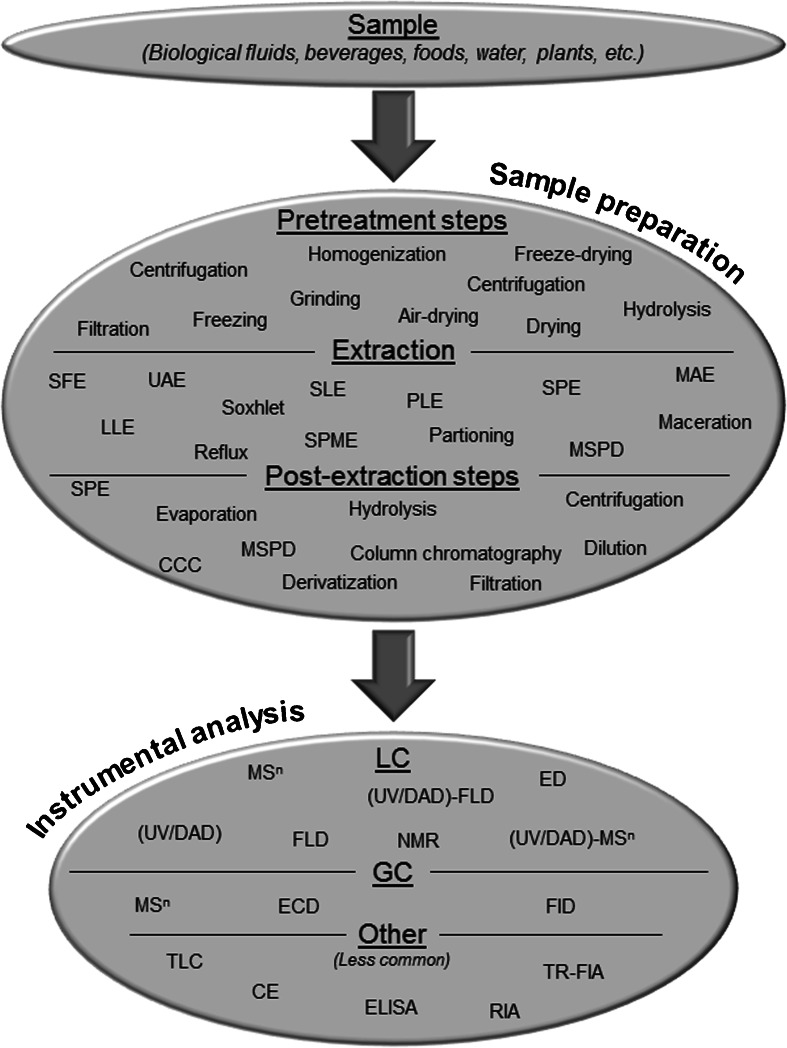
Main sample preparation and instrumental analysis methods for the determination of isoflavones and their related compounds. Abbreviations: *UAE* ultrasound-assisted extraction, *SFE* supercritical fluid extraction, *LLE* liquid-liquid extraction, *SLE* solid-liquid extraction, *SPME* solid-phase micro-extraction, *SPE* solid-phase extraction (incorporating the Quechers approach), *MAE* microwave-assisted extraction, *MSPD* matrix solid-phase dispersion, *CCC* counter-current chromatography, *LC* liquid chromatography (*HPLC* high-performance liquid chromatography, *UPLC* ultra-performance liquid chromatography, and *UHPLC* ultra high-performance liquid chromatography), *GC* gas chromatography, *MS* mass spectrometry, *UV* ultra-violet, *DAD* diode array detector (also called photodiode array detector, *PDA*), *FLD* fluorescence detector, *NMR* nuclear magnetic resonance, *ED* electrochemical detection, *ECD* electron capture detection, *FID* flame ionization detection, *TLC* thin layer chromatography, *CE* capillary electrophoresis, *ELISA* enzyme-linked immunosorbent assay, *RIA* radioimmunoassay, and *TR*-*FIA* time-resolved fluorescence immunoassay

In recent decades, due to the growing interest in isoflavones, a diverse range of analytical methods has been developed for isoflavone determination and quantification in plant material, soil, water, food, and food supplements, as well as in biological matrices, generating a huge amount of scattered information (Fig. 2). The overall analytical method used for isoflavones is highly dependent upon the matrix characteristics, the availability of the techniques, the desired selectivity, and the need to obtain information about the chemical structure of these compounds or simply to unambiguously identify previously targeted compounds. Initially, samples are usually freeze-dried or simply frozen. Depending on the matrix analyzed, isoflavones are extracted by classical maceration or liquid-liquid extraction (LLE), but a promising approach that is increasingly being used is to combine extraction/clean-up techniques such as ultrasound-assisted extraction (UAE), solid-phase extraction (SPE), microwave-assisted extraction (MAE) or matrix solid-phase dispersion (MSPD) (Rostagno et al. 2009). Given that isoflavones are often in conjugated forms, a hydrolysis step is sometimes used when the aim is to find aglycones. The number of analytical separation and detection techniques proposed is also numerous. Depending on the matrix and the information required, many of these techniques have been developed using variants of immunoassays (enzyme-linked immunosorbent assay, ELISA; radioimmunoassay, RIA; and time-resolved fluorescence immunoassay, TR-FIA), gas chromatography (GC), liquid chromatography (LC), and capillary electrophoresis (CE) coupled with various detection modes (mass spectrometry, MS; diode array detection, DAD; fluorescence detection, FLD; and electrochemical detection, ED) (Ko 2014; Rostagno et al. 2009; Valls et al. 2009; Vacek et al. 2008; Stalikas 2007; De Rijke et al. 2006; Grynkiewicz et al. 2005; Wu et al. 2004). Immunoassays are non-chromatographic methods with high sensitivity and specificity for single-component detection, high throughput screening and relatively low cost per sample. For this approach, however, preparing the antibodies for corresponding analytes is a challenging task and it suffers from the likelihood of cross-reactivity between the similar compounds, leading to a possible overestimation of the targeted analyte (Ko 2014; Wu et al. 2004). CE is also a promising technique with a high separation resolution and a low amount of sample required, but it is restricted to laboratories that have the necessary technical capacity (Wu et al. 2004; Wang et al. 2002). The most common analytical method used for quantifying isoflavones remains LC coupled with MS or ultra-violet detection (LC-MS, LC-UV). Thanks to its high sensitivity, MS detection is used preferentially when low amounts of isoflavones are thought to be in the matrix. UV detection is used when isoflavones are thought to be present in larger amounts (Ko 2014).

Milk is one of the most widely consumed foods in the world and several studies have been conducted in recent years to estimate its isoflavone content and to assess whether or not milk can be considered as a useful or dangerous source of isoflavones for humans. An initial answer was presented in two articles, especially with regard to the impact of cattle feed on the EQ content of milk (Kalač 2011, 2013). As far as we know, there has not yet been a specific review of the analytical methods used for quantifying isoflavones in cow’s milk. This paper, therefore, reviews the current situation with regard to sample preparation and analysis for the quantification of isoflavones in cow’s milk, including the most recent advances.

The literature overview was conducted online using the Scopus and CAB Abstracts bibliographic databases. Four keywords were identified: “phytoestrogens”, “isoflavones”, “milk”, and “quantitative analysis”. These keywords and their descriptors were used to create a Boolean equation in the two databases. The most recent studies (2005–2015) were selected. Key data of the selected articles are given in Tables 1 and 2. Where the authors of a selected article refer to a method published before 2005, the analytical protocol from the reference is summarized and presented in these tables. All the analytical procedures described in these articles were analyzed chronologically: initially, sample preparation procedures were compared and discussed, and then the instrumental analysis and validation parameters were examined.

**Table 1:** Summary of isoflavones analyzed, sample storage, and sample preparation methods used between 2005 and 2015 for quantification of isoflavones in milk

Isoflavone *(milk origin)*	Storage	Sample preparation			Reference
		Pre-extraction steps	Extraction	Post-extraction steps	
BA, DA, EQ, FO, GE, and PR (*feeding experiments and health impact experiments*)	Frozen (−20 °C)		- Deproteinized and defatted (2.5 mL milk with 0.25 mL acetate buffer [pH 5–5.2 and 2 mol.L^−1^], 1 mL heptane and 2 mL acetone) - Centrifugation - Evaporation to dryness (30 °C) - Reconstitution with 0.8 mL water	- Enz. hydrolysis (8 μL of β-glucuronidase/sulfatase from *H. pomatia* type H2, 40 °C, and 3–4 h) - 0.2 mL of MeOH was added - Centrifugation - Supernatant was analyzed	Adler et al. (2015), Adler et al. (2014), Njåstad et al. (2014), Höjer et al. (2012), Nielsen et al. (2012), Andersen et al. (2009a), and Steinshamn et al. ( 2008 ).
EQ (*commercial milks*)	Frozen (–18 °C)	- Thawed overnight at 4–6 °C, equilibrated to room T°, and homogenized (2 mL) - Enz. hydrolysis (0.1 mL β-glucuronidase/sulfatase from *H. pomatia* type H2 [≥45,000/3750 units.mL^−1^], 37 °C, and 2 h) - Cooled - Centrifugation	- Double L/L extraction (n-hexane and ethyl acetate, 3 × 3 mL) - Evaporation to dryness - Reconstitution MeOH:H_2_O (80:20, *v*/*v*)	- Filtration (0.2 μm)	Daems et al. (2015)
DA and GE (*commercial milks*)	Frozen (−20 °C) or directly analyzed		- Deproteinized (10 g of milk mixed with 12 mL of 1% (*v*/*v*) acetic acid in acetonitrile, 1 g NaCl, and 2.5 g MgSO_4_) - Centrifugation - Evaporation to dryness - Reconstitution (5 mL water)	- SPE (OASIS-HLB) - Evaporation - Reconstitution in 200 μL of H_2_O/MeOH (3:1, *v*/*v*) - Filtration (0.2 μm)	Wielogórska et al. (2015)
EQ (*technological processing*)	N.C.		- Deproteinized (10 mL of milk mixed with 3 mL of acetonitrile and centrifuged at 5 °C)	- Supernatant stored at –80 °C - Filtration (0.2 μm)	Tsen et al. (2014)
PU (*technological processing*)	Frozen (≤−10 °C)	- Freeze-drying - Defatted three times with hexane (1:5, *w*/*v*)	- Extraction with MeOH (1:3, *w*/*v*) - Evaporation to dryness - Reconstitution in MeOH (30 g.mL^−1^)	- Filtration (0.45 μm)	Rastogi et al. (2013)
GE and GE-in (*commercial milks*)	N.C.		- Double UAE (10 and 5 mL of acetonitrile, 15 min) - Centrifugation	- Filtration (0.45 μm) - Concentration and dilution - SPE (OASIS-HLB) - Concentration - Dilution	Maggioni et al. (2013)
DA, EQ, FO, and GE (*feeding experiments*)	Frozen (−18 °C)		- L/L extraction (20 mL milk/20 mL acetone/acetate buffer pH 5.2 [4:1, *v*/*v*]) - Centrifugation - Evaporation (halved)	- Enz. hydrolysis (0.2 mL sulfatase/glucuronidase from *H. pomatia*, 52 °C, and 2 h) - Centrifugation - SPE (C18) - Evaporation to dryness - Reconstitution in MeOH (80%, *v*/*v*) - Filtration (0.45 μm)	Flachowsky et al. (2011)
BA, DA, EQ, FO, and GL (*feeding experiments and health impact experiments*)	Frozen (−20 °C)	- Equilibration at room temperature - Enz. hydrolysis (β-glucuronidase, 100 μL.4 mL^−1^of milk, 37 °C, and 1 h) - Centrifugation (cream and precipitate were discarded)	- Double L/L extraction (hexane and ethyl acetate, 3 × 3 mL) - Evaporation to dryness - Re-suspension (0.5 mL acetonitrile)	- Filtration (0.2 μm)	Nielsen et al. (2011), Skaanild and Nielsen (2010), Nielsen et al. (2009) and Andersen et al. ( 2009b )
DA, EQ, GE, and GL (*technological processing and feeding experiments*)	Frozen (−20 °C)	- Enz. hydrolysis (100 μL β-glucuronidase [11,400 U.mL^−1^]/ sulfatase [3200 U.mL^−1^] from *H. pomatia* type H2 were added at 2 mL of milk and 3 mL of acetate buffer [0.1 M and pH 5], 37 °C, and 12 h)	- L/L extraction (3 × 2 mL ethylacetate) - Centrifugation - Evaporation to dryness - Reconstitution (0.5 mL methanol:water [1:1, *v*/*v*])		Křížová et al. (2011a), Křížová et al. (2011b), Krajčová et al. (2010), and Třináctý et al. ( 2009 )
BA, DA, EQ, FO, GE, and GL (*commercial milks*)	N.C.		- L/L extraction (10 mL milk/10 mL acetone/acetate buffer [2 M, pH 5.2, 9:1, *v*/*v*])	- Enz. hydrolysis (0.3 mL *H. pomatia* type H5 [500000 units. 20 mL^-1^ water], 52 °C, and 16 h) - Reverse-phase SPE (C18) - Normal phase SPE (SiOH)	Antignac et al. (2009)
BA, DA, EQ, FO, GE, and *O*-DMA (*commercial milks and feeding experiments*)	Frozen (−80 °C)	- Enz. hydrolysis (100 μL β-glucuronidase [500 units] and 80 μL sulfatase [40 units] were added at 5 mL milk, 37 °C, and 2 h)	- L/L extraction (1.5 mL ammonium acetate buffer 1.5 M + 7.5 mL diethyl ether, chilled at −80 °C, centrifugation, and repeated 3 times) - Evaporation to dryness (40 °C) - Reconstitution in 2 mL of sodium acetate buffer (0.2 M and pH 5) containing MeOH 20%	- Centrifugation - SPE (OASIS-HLB 3 cc) - Evaporation to dryness (60 °C) - Reconstitution 200 μL MeOH/sodium acetate buffer (pH 5, 0.2 M, 3:1, *v*/*v*) - Centrifugation - Filtration (0.45 μm)	Mustonen et al. (2009) and Hoikkala et al. ( 2007 )
BA, DA, EQ, FO, GE, and GL (*commercial milks*)	Frozen (−20 °C)	- Freeze-dried	- SLE (100 mg/2 mL of 10% MeOH in sodium acetate (0.1%, pH 5), three time)	- Enz. hydrolysis (purified β-glucuronidase from *H. pomatia*, cellulase from *Trichoderma reesi*, and β-glucosidase from almonds, 37 °C and 16 h) - SPE (Strata C-18E, 50 mg.mL^−1^) - Dried - Reconstitution (200 μL MeOH 40%)	Kuhnle et al. (2008)
DA, DA-in, GE, GE-in, GL, and GL-in (*technological processing*)	N.C.		- Deproteinized (2 mL of milk mixed with 50 μL IS and 3 mL acetonitrile and stored overnight at 10 °C)	- Equilibrated at room T° - Evaporation to dryness - Reconstitution (2.4 mL 50% acetonitrile) Filtration (0.22 μm)	Uzzan et al. (2007)

The underlined reference corresponds to the referenced method used and cited in the other articles

Abbreviations: *BA* biochanin A, *DA* daidzein, *DA*-*in* daidzin (glycoside form of DA), *EQ* equol, *FO* formononetin, *GE* genistein, *GE*-*in* genistin (glycoside form of GE), *GL* glycitein, *GL*-*in* glycitin (glycoside form of GL), *PR* prunetin, *PU* puerarin, and *O*-*DMA O*-desmethylangolensin

**Table 2:** Summary of separation and detection methods used between 2005 and 2015 for quantification of isoflavones in milk

Instrumental analysis		Validation parameters									Reference
Separation	Detection	Quantification mode	LOQ	LOD	Sel.	Lin.	Prec.	Acc.	Stab.	Rob.	
**HPLC** Column: Zorbax XDB (150 × 2.1 mm i.d., 3.5 μm) Mobile phases: (A) 0.5% acetic acid (B) MeOH Elution: gradient Column T°: N.C. Flow: 0.4 mL.min^−1^ Run time: 14 min Inj. vol.: 50 μL	MS/MS ES^+^, MRM mode	Standard addition	N.C.	0.05–0.17 ng.mL^−1^	N.C.	N.C.	N.C.	N.C.	N.C.	N.C.	Adler et al. (2015), Adler et al. (2014), Njåstad et al. (2014), Höjer et al. (2012), Nielsen et al. (2012), Andersen et al. (2009a), Steinshamn et al. ( 2008 )
**UPLC** Column: Acquity UPLC^®^ HSS T3 (100 × 2.1 mm, 1.8 μm) Mobile phases: (A) water/acetonitrile (95/5, *v*/*v*) with 0.01% formic acid (B) acetonitrile/water (95/5, *v*/*v*) with 0.01% formic acid Elution: gradient Column T°: 40 °C Flow: 0.6 mL.min^−1^ Run time: 5 min Inj. vol.: 10 μL	MS/MS ESI^+^, MRM mode	External calibration	5 ng.mL^-1^	0.3 ng.mL^−1^	V	V	V	V	V	V	Daems et al. (2015)
**UHPLC** Column: Acquity HSS T3 (100 × 2.1 mm, 1.8 μm) Mobile phases: (A) 5 mM ammonium acetate in H_2_O/acetonitrile (9:1, *v*/*v*) (B) MeOH:acetonitrile (1:1, *v*/*v*) Elution: gradient Column T°: 45 °C Flow: 0.4 mL.min^−1^ Run time: 10.5 min Inj. vol.: 10 μL	MS/MS ESI^±^, MRM mode	External calibration	0.35–0.36 µg.g^-1^	0.20–0.21 μg.g^−1^	V	V	V	V	V	N.C.	Wielogórska et al. (2015)
**LC**	IT-TOF-MS^−^	External calibration	N.C.	N.C.	N.C.	N.C.	N.C.	N.C.	N.C.	N.C.	Tsen et al. (2014)
**HPLC** Column: Supelcosil LC-8-DB (250 × 4.6 mm i.d., 5 μm) + guard column (40 × 4.6 mm i.d.) Mobile phases: (A) water (B) MeOH Elution: gradient Column T°: 25 °C Flow: 0.8 mL.min^−1^ Run time: 45 min Inj. vol.: 10 μL	PDA UV: 254 nm	External calibration	500 ng	300 ng	V	V	V	V	N.C.	N.C.	Rastogi et al. (2013)
**HPLC** Column: LUNA C8 (50 × 2.0 mm i.d., 5 μm) Mobile phases: (A) 0.05% acetic acid in Milliq water (B) acetonitrile Elution: gradient Column T°: N.C. Flow: 200 μL.min^−1^ Run time: 18 min Inj. vol.: 10 μL	MS/MS ES^−^, MRM mode	External calibration	N.C.	0.6–0.9 ng.g^−1^	N.C.	V	V	V	N.C.	N.C.	Maggioni_2013
**HPLC** Column: Purospher STAR RP-18^e^ (250 × 4.0 mm i.d., 5 μm particle size) Mobile phases: (A) aqueous 1% formic acid (B) acetonitrile 1% formic acid Elution: gradient Column T°: 35 °C Flow: 0.5 mL.min^−1^ Run time: 41 min Inj. vol.: 20 μL	DAD-MS/MS UV: 200–600 nm MS: ESI^+^, SIM mode	External calibration	0.006–0.7 ng.mL^-1^	N.C.	N.C.	V	N.C.	V	N.C.	N.C.	Nielsen et al. (2011) and Skaanild and Nielsen (2010), Nielsen et al. (2009) and Andersen et al. ( 2009b )
**HPLC** Column: YMC-Pack ODS-AM 12S05 column (150 × 4.6 mm i.d., 5 μm) Mobile phases: (A) acetic acid 0.1% (B) acetonitrile/acetic acid [99.9:0.1, *v*:*v*] Elution: gradient Column T°: N.C. Flow: 1.0 mL.min^−1^ Run time: 60 min Inj. vol.: N.C.	MS/MS ESI^−^, N.C. mode	N.C.	N.C.	N.C.	N.C.	N.C.	N.C.	N.C.	N.C.	N.C.	Flachowsky et al. (2011)
**HPLC** Column: discovery C18 (150 × 3 mm, 5 μm) + discovery C18 guard column (20 × 4 mm, 5 μm) Mobile phases: (A) MeOH (B) 0.1% acetic acid water (*v*/*v*) Elution: gradient Column T°: 45 °C Flow: 0.7 mL.min^−1^ Run time: 16 min Inj. vol.: 10 μL	MS/MS APCI^+^, SRM mode	External calibration	2–15 ng.mL^-1^	0.5–5 ng.mL^−1^	N.C.	N.C.	V	V	N.C.	N.C.	Křížová et al. (2011a), Křížová et al. (2011b), Krajčová et al. (2010) and Třináctý et al. ( 2009 )
**HPLC** Column: RP octadecyl grafted silica stationary phase Nucleosil® C_18_AB (50 × 2.1 mm, 5 μm + guard column 10 × 2.1 mm) Mobile phases: (A) methanol (B) water 0.5% acetic acid Elution: gradient Column T°: N.C. Flow: 0.3 mL.min^−1^ Run time: 30 min Inj. vol.: 10 μL	MS/MS ESI^+^, MRM mode	External calibration	0.05–0.50 ng.g^−1^	N.C.	V	V	V	N.C.	N.C.	V	Antignac et al. (2009)
**HPLC** Column: Zorbax Eclipse XDB-C18 (250 × 4.6 mm + precolumn Zorbax R-P 4 × 4 mm) Mobile phases: (A) MeOH/10 mM Na_2_HPO_4_.2H_2_O pH 6.5 (B) MeOH 100% Elution: gradient Column T°: 40 °C Flow: 1 mL.min^−1^ Run time: 31 min Inj. vol.: 10 μL	DAD-FLD UV: 260 nm FL: 254 nm (ex) 465 and 310 nm (em)	External calibration	3.3–117.2 ng.mL^-1^	1.3–58.6 ng.mL^−1^	V	V	N.C.	V	N.C.	N.C.	Mustonen et al. (2009) and Hoikkala et al. ( 2007 )
**HPLC** Column: diphenyl column (Varian Pursuit, 150 × 2.0 mm i.d., 3 μm) Mobile phases: (A) 40% ammonium acetate (0.1%, pH 4.8) in MeOH (B) 100% MeOH Elution: gradient Column T°: 50 °C Flow: 250 μL.min^−1^ Run time: N.C. Inj. vol.: N.C.	MS/MS ESI^−^, N.C. mode	External calibration	N.C.	15 ng.g^−1^ww	V	V	V	V	N.C.	N.C.	Kuhnle et al. (2008)
**HPLC** Column: C18 YMC-ODS-AM (250 × 3 mm S5) + C_18_ guard column (AJ0-7597) Mobile phases: (A) acetic acid: acetonitrile:water (0.1:9.95:89.95) (B) acetic acid:acetonitrile:water (0.1:89.95:9.95) Elution: gradient Column T°: 40 °C Flow: 0.8 mL.min^−1^ Run time: N.C. Inj. vol.: 10 μL	UV	External calibration	N.C.	N.C.	N.C.	V	N.C.	N.C.	N.C.	N.C.	Uzzan et al. (2007)

The underlined reference corresponds to the referenced method used and cited in the other articles

Abbreviations: *LOQ* limit of quantification, *LOD* limit of detection, *Sel*. selectivity, *Lin*. linearity, *Prec*. precision, *Acc*. accuracy, *Stab*. stability in extract or sample, *Rob*. robustness, *inj. vol*. injection volume, *N*.C. not communicated, and *V* communicated

## Sample preparation

Broadly, an analytical method can be split into two distinct parts: sample preparation and sample analysis. Sample preparation is undertaken before instrumental analysis and should ensure that: (1) the target compounds can be extracted from the matrix; (2) interference that could disrupt the detection of the target analyte or damage the chromatographic column can be eliminated or reduced; (3) to allow analysis of samples with low concentrations of the target compound; and (4) the analyte of interest can be transformed into a more suitable form that could be easily separated, detected and quantified. In Table 1, all the information about sample preparation found in the 26 selected articles is detailed and split into four chronological steps. The table also presents the origin of the milk and the isoflavones quantified. Studies using the same sample preparation procedure were grouped together. Articles where the sample preparation procedure was based on a previous study, but with some modifications, were not grouped (Daems et al. 2015; Flachowsky et al. 2011; Antignac et al. 2009). Thirteen sample preparation procedures were identified and are discussed in the next section.

### Sample conditioning

As shown in Table 1, most milk samples in the experiments reviewed were simply frozen between the sampling and analysis days. In other articles, sample condition was not mentioned. Often, information about the handling of milk samples after collection and before actual analysis was limited. An exception was Wielogórska et al. (2015), who reported that isoflavones (DA and GE) were stable in the matrix for only 2 weeks, indicating that all samples were analyzed immediately after collection or stored at −20 °C for no longer than 2 weeks before being thawed for analytical purposes. Poor storage can lead to errors that cannot be corrected later and that will affect the outcome of the final analysis. How should samples be stored, and for how long, without affecting their original isoflavone profile? For matrices such as soybean, some isoflavones (mainly the conjugated forms) have a fairly unstable character (Rostagno et al. 2009). In ruminants, glucuronide-conjugated compounds are the major form found in biological fluids (Mostrom and Evans 2012; Nielsen et al. 2012). The degradation problem could, therefore, also arise during milk storage and the information about the conjugated form content could be lost. More attention should be given to the storage and handling of milk samples between sampling and analysis in future studies.

If freeze-drying is an easy method for milk-sample storage, it would be interesting to check isoflavone stability under a range of temperature and storage-time conditions. The freezing method could be also compared with other techniques, such as freeze-drying. Isoflavones can be found in powdered milks (Maggioni et al. 2013; Antignac et al. 2009). Therefore, freeze-drying, which is often used for plant matrices because it does not seem to affect these compounds (Hoerger et al. 2011), could be an interesting alternative to the traditional freezing process. These findings need to be taken into account in future studies.

### Hydrolysis

Among the 13 sample preparation procedures listed in Table 1, only four did not include a hydrolysis step. Wielogórska et al. (2015) proposed a method without a hydrolysis step for the quantification of 19 endocrine disruptors in milk, including DA and GE. Rostagi et al. (2013) did not use a hydrolysis step because they sought to quantify puerarin (PU, the 8-C-glucoside of daidzein) from pasteurized toned milk enriched with extracts from *Pueraria tuberosa* Linn (commonly called “kudzu”). According to these authors, this glycoside form of isoflavone that is abundant in kudzu might be beneficial in the treatment and prevention of some diseases and cancers. The two other procedures were used by Maggioni et al. (2013) and Rastogi et al. (2013) to quantify aglycones in infant powdered milk formulas and glycosides in soy-fortified milk. Integrating a hydrolysis step into the analytical protocol depends on the objectives sought. Hydrolysis is often used when the exact nature and composition of isoflavone glycosides in the matrix analyzed are unknown or when target aglycone compounds are sought. It allows the time required for instrumental analysis to be considerably reduced and facilitates the separation of target compounds. It also makes it unnecessary to use standard references that are difficult to obtain, not available, or often expensive. Introducing an additional step, however, can increase analytical variability.

As shown in Table 1, most of the studies reviewed used a hydrolysis step. Unlike other matrices where three procedures are reported (enzymatic, acidic and, least common, basic hydrolysis) (Rostagno et al. 2009; Schwartz et al. 2009), for milk only enzymatic hydrolysis is carried out to hydrolyze isoflavones. The authors focused on aglycones because these forms appear to be the most biologically active and can be absorbed by the intestinal tract of humans and animals (Ko 2014; Mostrom and Evans 2012). In addition, some isoflavones, such as EQ, seem to be sensitive to acidic conditions (Setchel and Clerici 2010). Enzymatic hydrolysis involves incubating the sample with enzymes at a fixed temperature (within a range of 37 to 52 °C, depending on the study) and over different incubation times (between 1 and ≥16 h). A solution buffer (pH of about 5) is sometimes used to optimize hydrolysis conditions. Unlike plant matrices (Rostagno et al. 2009), only β-glucuronidase/sulfatase from *Helix pomatia* was involved in the hydrolysis of isoflavones in milk. This enzymatic juice contains glucuronidase and sulfatase activities. The former catalyzes the breakdown of complex carbohydrates (glucuronides) and the latter catalyzes the hydrolysis of sulfate esters, the two main forms of isoflavones found in biological fluids (Ko 2014; Nielsen et al. 2012; Rostagno et al. 2009). Only Kuhnle et al. (2008) reported using a mixed solution of three enzymes: β-glucuronidase from *H. pomatia*; cellulase from *Trichoderma reesi*; and β-glucosidase from almonds.

The hydrolysis temperature varied between 37 and 40 °C, but it did reach 52 °C in some studies (Flachowsky et al. 2011; Antignac et al. 2009). The temperature generally used by suppliers to evaluate the glucuronidase and sulfatase activities from these enzymes was 37 °C. Where a buffer solution was used, the pH of the solution varied between 4.6 and 5.2. The optimal pH values given by suppliers were 5.0 and 6.2 for glucuronidase and sulfatase activities, respectively. Hydrolysis time varied, ranging from 1 h (Andersen et al. 2009b) to overnight (Antignac et al. 2009; Kuhnle et al. 2008). During an evaluation of the robustness of their hydrolysis protocol based on a one-variable-at-a-time technique, Daems et al. (2015) demonstrated that an incubation time of 1 h was sufficient for hydrolyzing conjugated forms of EQ. Another important point, but one on which there is little information in the studies, is the minimum enzyme activity needed to hydrolyze all the conjugated forms of isoflavones present in milk. When enzyme activity values were provided by authors or when estimating them was possible, it was found that they varied greatly across the studies. For example, for their analysis of isoflavones (containing EQ) in commercial milk, Hoikkala et al. (2007) used 100 units of glucuronidase activity per ml of sample, whereas Daems et al. (2015) used 2250 units. In the product information data sheets for other biological fluids, Sigma-Aldrich recommends using between 1000 and 20,000 units per mL of β-glucuronidase type H2 from *H. pomatia* (EC 3.2.1.31), but no specific information is given on the hydrolysis of milk samples. One disadvantage of using *H. pomatia* juice is that it usually contains appreciable levels of some isoflavones (according to Taylor et al. (2005): 13.38 ng GE.mL^−1^ enzyme, 0.25 ng DA.mL^−1^, and 0.58 ng EQ.mL^−1^) which can affect the quantification. Daems et al. (2015) and Grace and Teale (2006) did not detect any trace of EQ, and Grace and Teale (2006) found that GE was the major isoflavone in *H. pomatia* juice. These observations show that isoflavone concentrations appear to fluctuate from one enzyme batch to another. The hydrolysis with this enzymatic solution also showed interfering peaks with isoflavone signals in the chromatogram of human milk extracts (Franke and Cluster 1996). There are two ways of addressing these problems. One is a pre-cleanup of the enzymatic preparation by SPE (Kuhnle et al. 2007; Grace and Teale 2006). The second way, probably the easier of the two, involves working with a blank sample where the milk has been replaced by a buffer solution in order to estimate the isoflavone content in the enzymatic mixture (Taylor et al. 2005).

### Isolation of target compounds

Solvent extraction is the main step used for the recovery and isolation of bioactive compounds from a matrix before instrumental analysis and it can be performed before or after the hydrolysis step. Additional steps are sometimes necessary to remove lipids or proteins, which can cause interferences during instrumental analysis. Fat is one of the major causes of the matrix effect in LC-MS (Jiang et al. 2012; Trontelj 2012) and most of the protocols in Table 1 therefore indicate defatting and deproteinizing steps. Lipids can be removed by LLE with an apolar solvent (e.g., n-heptane or n-hexane) and centrifugation. Proteins are removed by precipitation in acidic conditions and centrifugation. Deproteinization is often used at the same time that the defatting or extraction of target compounds is done because organic solvents can also precipitate the proteins (Trontelj 2012).

Liquid-liquid extraction is the most widely used technique for extracting isoflavones from milk. The extractions are repeated two to three times and the extracts are pooled. Among the analytical protocols listed in Table 1, only three used SLE to recover isoflavones in milk samples (Maggioni et al. 2013; Rastogi et al. 2013; Kuhnle et al. 2008). Only Rastogi et al. (2013) deliberately used the freeze-drying process, whereas two other studies used this extraction technique because they were analyzing milk samples in powder form. Optimizing extraction is a challenging task given the wide polarity range of isoflavones. As an example, compared with DA, GE has an additional hydroxyl group and therefore DA and GE are expected to have different solubilities (Harjo et al. 2007). In general, conjugated forms are more polar than aglycones and are extracted with polar solvents such as alcohols (MeOH, EtOH) or acetonitrile, often mixed with varying proportions of water. For isoflavone quantification in milk, a hydrolysis step is almost always used in order to remove the polar groups from conjugated forms, leading to the formation of isoflavone aglycones, which are less polar and almost insoluble in water (Vitale et al. 2013; Ishii et al. 2013; Harjo et al. 2007). This is why less polar solvents (diethyl ether or ethyl acetate), with no water added, are used for extraction in milk. The choice of solvent composition should be determined empirically according to the target molecules that the researchers wish to extract.

Looking more closely at the order in which the hydrolysis and isolation steps are done, some authors conducted the hydrolysis step first and then the extraction step, whereas others used the reverse order. This raises the question: is one protocol better than the other? If the less polar solvents are more efficient for extracting aglycone forms, when the hydrolysis is performed after the extraction step the conjugated isoflavones might not be completely extracted, leading to an underestimation of the overall isoflavone content. Some authors (Daems et al. 2015; Křížová et al. 2011a) reported that isoflavones are distributed more widely in the aqueous fraction than in the lipid fraction. In contrast, Tsen et al. (2014) reported that isoflavones are distributed mostly in the lipid fraction and that the defatting process, therefore, reduces the overall amount of isoflavones in a sample. These are all matters that need to be addressed in future studies.

### Purification and concentration extracts

After the extraction of isoflavones in milk, the extract is subjected to a series of post-treatment steps before instrumental analysis. In almost all protocols, centrifugation is performed. As well as eliminating the cream (fat) and the precipitated proteins, it also isolates other small solid impurities. A filtration step is often performed just before the sample extract injection (Wielogórska et al. 2015; Daems et al. 2015; Tsen et al. 2014; Rastogi et al. 2013; Flachowsky et al. 2011; Andersen et al. 2009b; Nielsen et al. 2009; Hoikkala et al. 2007; Uzzan et al. 2007) to prevent damage to the analytical columns.

In milk, isoflavones are usually present in low concentrations (a few ppb or, sometimes, higher for microbial metabolites) (Kalač 2013) and, therefore, it is necessary to concentrate the target analyte before instrumental analysis. SPE and evaporation were the most commonly used enrichment techniques. SPE using C_18_ bonded silica (Flachowsky et al. 2011; Antignac et al. 2009; Kuhnle et al. 2008) or a universal polymeric reversed-phase sorbent called OASIS-HLB from Waters (Wielogórska et al. 2015; Maggioni et al. 2013; Mustonen et al. 2009; Hoikkala et al. 2007) were reported to provide a clean concentrated isoflavone extract. Probably due to its typical chemistry, OASIS-HLB has the advantage of greater retention and good recoveries even if the sorbent runs dry (Rostagno et al. 2009). The final extracts are then evaporated until dry and re-suspended with the polar solvent used in the instrumental analysis.

## Instrumental analysis

After these preparation steps, the sample extract can be injected into instruments that will allow the target compounds to be separated, detected and quantified. The data in the 26 selected articles are presented in Table 2. When different authors used the same analytical methods, the data presented are derived from all the information in the articles. Thirteen analytical methods were identified and are discussed below.

### Analyte separation

Unlike in other matrices (plants, foods or other biological fluids), there are few separation methods reported in the literature for the quantification of isoflavones in milk. Over the past 10 years, the only analytical separation technique used has been reverse-phase LC with a binary solvent system that contains acidified water and an acidified polar organic solvent, such as methanol or acetonitrile (Table 2).

With the LC technique, a guard column is often placed before the analytical column in order to retain all impurities remaining in the sample extract (Rastogi et al. 2013; Křížová et al. 2011a, b; Antignac et al. 2009; Třináctý et al. 2009; Hoikkala et al. 2007; Uzzan et al. 2007). This also enables the compounds from the extract to start being separated. These small columns are regularly changed and allow the lifetime of the main analytical column to be increased. They often have a stationary phase similar to that of the main column. Columns chosen for isoflavone separation in milk are exclusively packed with reversed-phases, mainly C_18_ with an internal diameter of 2.0–4.6 mm, a length of 50–250 mm, and a particle size of 3–5 μm. Two other stationary phase chemistries are sometimes also mentioned in the articles: C_8_ (Rastogi et al. 2013; Maggioni et al. 2013) and diphenyl (Kuhnle et al. 2008). Two recent studies report on the use of ultra high-performance LC (UHPLC) columns with a particle size of 1.8 μm, an internal diameter of 2.1 mm, and a length of 100 mm (Wielogórska et al. 2015; Daems et al. 2015). This technology allows separations that have positive effects on both resolution and accuracy. It also increases sample-throughput due to reduced analysis times (Gumustas et al. 2013; Valls et al. 2009). The reduction of analysis time is illustrated in Table 2. For classical LC analysis, the average analysis time is about 35 min, ranging from a minimum of 14 min (Steinshamn et al. 2008) to a maximum of 60 min (Flachowsky et al. 2011). In the two studies using UHPLC it was between 5 and 10.5 min. These times could be further reduced by manipulating the column temperature, flow rate, mobile phase composition, and solvent gradients.

The mobile phase flow rate depends upon its composition, column type and temperature. In the selected articles, the flow rates varied between 0.2 and 1 mL.min^−1^, the temperature from 25 to 45 °C, and the injection volume from 10 to 50 μL. The mobile phase is usually a mixture of water and an organic solvent (methanol or acetonitrile) with a small proportion of formic or acetic acids (up to a max. of 1%). The differing hydrophobicities of isoflavones indicate that the gradient elution mode should be used (Rostagno et al. 2009). Since isoflavones exhibit a weak acidic nature, the use of acids in the mobile phase can enhance chromatographic resolution and peak shape (Rostagno et al. 2009; Wu et al. 2004). Acidification of the mobile phase also significantly increases the isoflavone limit of detection (LOD) by promoting positive ion formation in the MS source (Wu et al. 2004).

### Analyte detection

As shown in Table 2, the most commonly used detection methods combined with LC are MS and UV detectors. They can be used alone, in combination (Andersen et al. 2009b; Nielsen et al. 2009) or with other types of detectors (Mustonen et al. 2009; Hoikkala et al. 2007). Hoikkala et al. (2007) used UV-DAD at 262 nm for GE, *O*-DMA and BA and they used FLD for DA and FO (λ_ex_ at 254 nm and λ_em_ at 465 nm), as well as for EQ (λ_ex_ at 254 nm and λ_em_ at 310 nm). Although the LC-FLD method is often more sensitive than the UV absorption methods, the number of isoflavones that are naturally fluorescent is fairly limited (Ko 2014). In addition, the acidification of the mobile phase has a quenching effect that can reduce FLD sensitivity (Vacek et al. 2008).

Overall, LC-UV and LC-MS are the two main detection modes used for quantifying isoflavones. In milk, however, MS is preferred to UV detection. UV detection uses the isoflavone characteristic of having at least one aromatic ring that strongly absorbs UV light, with a maximum wavelength ranging from 230 to 280 nm (Ko 2014; Rostagno et al. 2009; Stalikas 2007; Wu et al. 2004). A range of 300 to 550 nm was sometimes also used as a second maximum wavelength and attributed to the substitution pattern and conjugation of the C-ring (Stalikas 2007). This is not the case with all isoflavones. For example, the microbial metabolites EQ and *O*-DMA exhibit poor UV absorption characteristics, making UV detection unsuitable for their measurement (Ko 2014; Setchel and Clerici 2010). For isoflavones that exhibit this UV absorption characteristic, the UV–visible spectra of many of them are very similar (Vacek et al. 2008). Another disadvantage of this method is its low sensitivity, which restricts its use to quantification in matrices likely to have a large amount of isoflavones, such as plants, plant-derived samples and supplemented foods (Ko 2014; Wu et al. 2004). Among the studies presented in Table 2, only two used UV as the sole mode of detection (Rastogi et al. 2013; Uzzan et al. 2007) and both focused on analyzing fortified milk.

In order to overcome the problems of non-specificity and lack of sensitivity, researchers have focused on MS detection. Usually, this method is highly sensitive, with low limits of quantification (LOQ) under or around the ppb level (see Table 2). Electrospray ionization (ESI) and atmospheric pressure chemical ionization (APCI) are the two sources most commonly used for quantifying isoflavones in milk. In the selected articles, these sources were used in positive and/or negative mode, except for APCI, which was used exclusively in positive mode with select reaction monitoring (SRM). In the ESI, multiple reaction monitoring (MRM) was used. ESI and APCI interfaces appeared to be suitable for the analysis of isoflavones, but ESI is better suited for the conjugated forms. ESI is the softest and most suitable method for ionization without the in-source fragmentation of the relatively weak glucuronide ether or ester bonds. Thermally labile conjugates form molecular ions in ESI, but with APCI these conjugates decompose because they are not stable enough (Trontelj 2012; Hoikkala et al. 2003). In their comparison of ESI and APCI sources for the quantitative determination of phytoestrogens in urine, Rybak et al. (2008) showed that, for EQ, ESI was better in terms of measurement precision, sensitivity and LOD.

The benefits of LC-MS include high precision and sensitivity, suitability for determining isoflavones and their metabolites or conjugated forms, less samples manipulation than for GC, and applicability to non-volatile analytes with direct injection of the liquid samples. The higher price of this technology remains its major disadvantage compared with other separation and detection technologies. LC-MS, however, is now a fast-moving field, with many new detection and chromatographic techniques being developed. With their shorter analysis time, lower solvent consumption, better resolution properties, and better sensitivity than classical LC, UPLC and UHPLC with MS appear to be the fastest and easiest methods to implement and to produce reliable results.

### Validation parameters

As far as we could ascertain, method validations were seldom discussed in the previous review articles on isoflavones and related compounds. Where they were mentioned, the validation parameters are shown in Table 2 (when several works used the same analytical procedure, the various data in each study were collected and summarized in this table).

The validation parameters taken into account in this table come from the EMA (2015) guidelines, which describe the suitable criteria for the validation of analytical methods used in veterinary drug residue depletion studies. The guidelines could apply to the quantification of isoflavones in milk because these estrogenic compounds can be active in very low amounts. As shown in Table 2, most of the studies used external calibration as the quantification mode for determining the isoflavone content of milk, whereas Steinshamn et al. (2008) and related studies used a standard addition protocol. Among these studies, Andersen et al. (2009a) reported that the LOD for FO and EQ were 0.047 and 0.170 ng.mL^−1^, respectively. In the other selected articles, the validation parameters most often omitted were sample or extract stability and the robustness of the analytical method proposed. The most comprehensive validation procedures were reported by Wielogórska et al. (2015) (for DA, GE and other endocrine disruptors) and Daems et al. (2015) (for EQ).

## Conclusion

This paper summarizes the current analytical methods used for quantifying isoflavones and their metabolites in cow’s milk. Careful examination of methodological aspects showed that most of the studies used LLE to extract isoflavones from milk. Given that aglycones are the most biologically active forms of isoflavones, most of the analytical protocols used a hydrolysis step with β-glucuronidase/sulfatase from *H. pomatia*.

Although non-chromatographic immunoassays and CE with MS or ED detectors are seen as promising techniques for quantifying isoflavones, reverse-phase LC-MS is the only analytical method used in the past 10 years. LC-MS is now a fast-moving field, with many new detection and chromatographic techniques being developed over the past decade that achieve high sensitivity and reduce analysis time. The low isoflavone content (about ppb value) naturally present in milk, except in the case of some isoflavone metabolites, justifies the use of MS as a detection method. The new chromatographic technologies, UPLC and UHPLC, reduce the time needed and achieve good resolution.

Although some analytical protocols have been used frequently by researchers, there is still no standardized procedure available for the sample preparation and determination of these phytoestrogenic compounds in milk. A rigorous validation referring to a guideline would be enough to ensure the reliability of the chromatographic results. For sample preparation, however, a standardized procedure is interesting to compare the results from the different studies.
